# Preclinical Development of a Therapy for Chronic Traumatic Spinal Cord Injury in Rats Using Human Wharton’s Jelly Mesenchymal Stromal Cells: Proof of Concept and Regulatory Compliance

**DOI:** 10.3390/cells11142153

**Published:** 2022-07-08

**Authors:** Joaquim Vives, Joaquim Hernández, Clémentine Mirabel, Maria Puigdomenech-Poch, David Romeo-Guitart, Sara Marmolejo-Martínez-Artesero, Raquel Cabrera-Pérez, Jessica Jaramillo, Hatice Kumru, Joan García-López, Joan Vidal-Samsó, Xavier Navarro, Ruth Coll-Bonet

**Affiliations:** 1 Banc de Sang i Teixits, Edifici Dr. Frederic Duran i Jordà, Passeig Taulat 116, 08005 Barcelona, Spain; jvives@bst.cat (J.V.); clementine.mirabel@gmail.com (C.M.); rcabrera@bst.cat (R.C.-P.); joangarcia@bst.cat (J.G.-L.); 2 Musculoskeletal Tissue Engineering Group, Vall d’Hebron Research Institute (VHIR), Universitat Autònoma de Barcelona, Passeig de la Vall d’Hebron 129-139, 08035 Barcelona, Spain; 3 Departament de Medicina, Universitat Autònoma de Barcelona, Passeig de la Vall d’Hebron 129-139, 08035 Barcelona, Spain; hkumru@guttmann.com (H.K.); jvidal@guttmann.com (J.V.-S.); 4 Institute of Neurosciences, Department of Cell Biology, Physiology and Immunology, Facultat de Medicina, Universitat Autònoma de Barcelona, Av. Can Domènech, Edifici M, Campus UAB, 08193 Bellaterra, Spain; joaquim.hernandez@uab.cat (J.H.); mariapuig.poch@gmail.com (M.P.-P.); romeoguitart@gmail.com (D.R.-G.); smarmoma@gmail.com (S.M.-M.-A.); jesica.jaramillo@uab.cat (J.J.); 5 Fundación Institut Guttmann, Institut Universitari de Neurorehabilitació, Universitat Autònoma de Barcelona, 08916 Badalona, Spain; 6 Fundació Institut d’Investigació en Ciències de la Salut Germans Trias i Pujol, 08916 Badalona, Spain

**Keywords:** mesenchymal stromal cells, translational medicine, cell therapy, stem cells, cell-based therapy, spinal cord injury, animal model, preclinical research, advanced therapy, good laboratory practice

## Abstract

(1) Background: the use of Mesenchymal Stromal Cells (MSC) in emerging therapies for spinal cord injury (SCI) hold the potential to improve functional recovery. However, the development of cell-based medicines is challenging and preclinical studies addressing quality, safety and efficacy must be conducted prior to clinical testing; (2) Methods: herein we present (i) the characterization of the quality attributes of MSC from the Wharton’s jelly (WJ) of the umbilical cord, (ii) safety of intrathecal infusion in a 3-month subchronic toxicity assessment study, and (iii) efficacy in a rat SCI model by controlled impaction (100 kdynes) after single (day 7 post-injury) and repeated dose of 1 × 10^6^ MSC,WJ (days 7 and 14 post-injury) with 70-day monitoring by electrophysiological testing, motor function assessment and histology evaluation; (3) Results: no toxicity associated to MSC,WJ infusion was observed. Regarding efficacy, recovery of locomotion was promoted at early time points. Persistence of MSC,WJ was detected early after administration (day 2 post-injection) but not at days 14 and 63 post-injection. (4) Conclusions: the safety profile and signs of efficacy substantiate the suitability of the presented data for inclusion in the Investigational Medicinal Product Dossier for further consideration by the competent Regulatory Authority to proceed with clinical trials.

## 1. Introduction

Spinal Cord Injury (SCI) is a devastating condition resulting in loss of sensory and motor functions [1]. Symptomatology varies widely depending on the level and/or the severity of the injury. Current treatment strategies involve mechanical stabilization of the column and management of local inflammation to prevent further damage [2]. However, the absence of any therapy to reverse spinal cord damage have encouraged the development of novel approaches based on the use of stem cells. Indeed, cell therapy has received considerable attention in the treatment of traumatic injuries that affect the spinal cord, being the transplantation of mesenchymal cells the main focus in this area [3,4]. 

Multipotent Mesenchymal Stromal cells (MSC) constitute a defined population of cells with both paracrine activity and differentiation potential into mesenchymal lineages that can be readily derived ex vivo in culture from virtually all vascularized tissues in the body, being bone marrow, the umbilical cord and lipoaspirates the most common tissue sources [3,5]. Regardless of their tissue of origin, MSC express and secrete multiple bioactive molecules involved in anti-inflammatory, immunomodulatory, neurotrophic and angiogenic pathways, which can promote neuroprotection (i.e., mitigation of secondary damage, oligodendrocytes and neuronal degeneration), neuroregeneration (i.e., axon and myelin regeneration) and neuroplastic changes (i.e., axon sprouting, neuronal reconnection) [4,6]. Indeed, the immunomodulatory and anti-inflammatory paracrine activities of MSC are of therapeutic interest in a number of pathologies, including SCI [7,8,9].

According to preclinical data in rodents, injection of MSC at early stages following SCI may (i) prevent progression of tissue damage and improve deficits, (ii) correlate with improved endpoints in the follow-up, and (iii) reduce demyelination and loss of axons by secretion of neuroprotective and pro-oligodendrogenic molecules that prompt tissue sparing [10,11].

In the context of a pilot Phase I/IIa, double-blind, randomized clinical trial, we recently reported that a single dose of allogeneic MSC derived from the Wharton’s jelly of the umbilical cord (MSC,WJ) administered intrathecally is safe and induces clinical effects, although limited to sensory improvement in the segments adjacent to the spinal injury in patients with chronic, complete traumatic SCI at thoracic level [12]. These promising results encouraged us to evaluate safety and efficacy of repeated dosing of MSC,WJ. Prior to clinical testing, however, we (i) investigated single and repeated administration of MSC,WJ in a rat model of traumatic SCI and (ii) assessed local and systemic toxicity of intrathecal administration of MSC,WJ in immunosuppressed rats, to complete the preclinical package of the existing Investigational Medicinal Product Dossier (IMPD) in compliance with current regulatory guidelines and pharmaceutical quality management standards [13].

## 2. Materials and Methods

### 2.1. Study Design as Part of the Product Development Programme

The present study covers pharmacodynamics (PD), pharmacokinetics (PK) and toxicology (Tox) aspects of intrathecal administration of MSC,WJ, in the context of a broader non-clinical development package (described in Table 1) included in the IMPD submitted to the competent regulatory authority with the aim of clinical testing a candidate advanced therapy for SCI [14,15]. All animal procedures were conducted in accordance with local, national and European legislation (Decret 214 de 1997, Real Decreto 53 de 2013, European Directive 2010/63/EU, respectively) and were approved by the Universitat Autònoma de Barcelona’s Ethical Committee on Human and Animal Experimentation.

### 2.2. Cell Cultures

Clinical grade ex vivo expanded MSC were derived from the Wharton’s jelly of human umbilical cords following current Good Manufacturing Practice (GMP)-compliant methods reported in detail elsewhere [16,17]. Cells were expanded in vitro up to sufficient numbers (≤5 passages) for further testing by using Dulbecco’s Modified Eagle’s Medium (DMEM; Gibco) containing 2 mM glutamine supplemented with 10% human serum (hSer) B [9,16]. All cultures were maintained at 37 °C and 5% CO_2_ in humidified incubators. Cell number and viability were determined by the hemocytometer-based Trypan blue dye exclusion method in duplicates and the average value was calculated.

### 2.3. Cell Proliferation Assay

Proliferation of MSC,WJ cultured in presence or absence of FK506 (Fujisawa Pharmaceuticals, Osaka, Japan) was monitored by using the ATP-based CellTiter-Glo^®^ Luminescent Cell Viability Assay (Promega, Madison, WI, USA) as reported elsewhere [18]. Briefly, cells were cultured in multiwell format plates in the presence or absence of 10 ng/mL FK506 diluted in DMSO and, at the time of analysis, luminescent reagent was added to each well at 1:1 (*v*/*v*) ratio with respect to culture medium and mixed for 2 min using an orbital shaker. After 15 min incubation in the dark, 100 µL of supernatant from each well were transferred into opaque-walled 96 well-plates and luminescence was measured in triplicates on a Triad Multimode detector plate reader with Concert Triad Series software v2.1 (Dynex Technologies, Chantilly, VI, USA).

### 2.4. Flow Cytometry

Flow cytometric analysis was performed to evaluate expression of cell surface markers using 1:20 dilution of the panel of antibodies described next: CD31 (WM59, BD Pharmingen ref. 555445), CD45 (HI30, BD Pharmingen ref. 555482), CD73 (AD2, BD Pharmingen ref. 550257), CD90 (F15-42-1-5, Beckman Coulter ref. IM1839U), CD105 (43A4E1, Miltenyi Biotec ref. 130-094-941) and HLA-DR (L243, BD Biosciences ref. 347400) in a FACSCalibur device with CellQuest Pro software v5.1 (Becton Dickinson, Franklin Lakes, NJ, USA). Each sample was analysed twice and results were reported as the average value. Validation of the methods for assessing cellular identity was reported elsewhere [19].

### 2.5. Lymphocyte Proliferation Assay

The immunomodulation potency of MSC,WJ was determined by their capacity to inhibit the proliferation of polyclonally stimulated lymphocytes in vitro, using optimized methods as comprehensively described elsewhere [8]. Briefly, nucleated cells (NC) were obtained by density gradient (Histopaque-1077; Sigma-Aldrich, Saint Louis, MO, USA) from 24- to 48-h-old peripheral blood of healthy donors, which were confirmed negative for hepatitis B virus (HBV), hepatitis C virus (HCV), human immunodeficiency virus (HIV), and syphilis, both by serology and viral nucleic acid detection (NAD). Next, 2.5 × 10^6^ NC/mL were labelled with 0.625 μM carboxy–fluorescein diacetate succinimidyl ester (CFSE) for 10 min using the CellTrace™ CFSE Cell Proliferation Kit (Molecular Probes, Eugene, OR, USA). After washing, 2 × 10^7^ cells/mL were incubated for 12 min at 37 °C, washed again and seeded onto flat-bottomed 96-well plates (Corning, Corning, NY, USA) at an NC:MSC 5:1 ratio. Activation of lymphocytes was carried out with 25 ng/mL Phorbol 12-myristate 13-acetate (PMA, Sigma-Aldrich) and 0.5 μM Ionomycin (Sigma-Aldrich) in a final volume of 0.5 mL/well of “expansion medium”. Proliferation of NC was determined by measuring the reduction of fluorescence intensity at day 5 using flow cytometry (FACSCalibur, Becton Dickinson), and data were analysed with the FlowJo software v10 (TreeStar Inc., Ashland, OR, USA).

### 2.6. Subchronic In Vivo Toxicity and Biodistribution Study

Toxicity and biodistribution of human MSC,WJ were evaluated in 7- to 8-weeks old NIH nude immunodeficient male rats (Charles River Laboratories, France). To this end, either 20 μL of a saline solution (Plasmalyte 148, Baxter, Deerfield, IL, USA) containing 2% of human serum albumin (Albutein, Grifols, Barcelona, Spain) (control group, n = 3) or 20 μL of a saline solution containing 2% of human serum albumin and 1 × 10^6^ MSC,WJ (treated group, n = 9) were injected intrathecally. Accurate clinical evaluation was carried out for 3 months. At the end of the experimental period (day 84), animals were sacrificed by an intraperitoneal overdose of sodium pentobarbital and a complete macroscopic necropsy consisting of the assessment of natural orifices, fur, skin, musculature, thoracic cavity, mediastinum, and abdominal cavity was performed. Additionally, brain, heart, lungs, trachea, oesophagus, stomach, liver, spleen, pancreas, kidneys, salivary glands, small and large intestine, lymph nodes, spinal cord, and genitourinary system were meticulously examined, extracted and processed for histological studies. To analyse the biodistribution and persistence of the injected MSC,WJ, the human *CART1* gene sequence was amplified by semiquantitative end-point PCR in gonads, kidney, spleen, lung, and liver, as reported elsewhere [20]. This study was carried out in accordance with the Organisation for Economic Co-operation and Development (OECD) Principles of Good Laboratory Practice, as revised in 1997, C(97) 186/Final (Paris, France), Directive 1999/11/CE 8 (EU), and Real Decreto 1369/2000 (Spain).

### 2.7. Spinal Cord Injury Contusion Model

Adult female Sprague-Dawley rats (9 weeks old; 250–300 g) were housed with free access to food and water at room temperature of 22 ± 2 °C under a 12:12 light-dark cycle. Under anesthesia with ketamine (90 mg/kg) (Imalgene^R^ 1000; Boehringer Ingelheim, Rhein, Germany) and xylazine (10 mg/kg) (Rompun^R^; Bayer, Leverkusen, Germany) and aseptic conditions, a longitudinal dorsal incision was made to expose T6-T10 spinous processes. A laminectomy of T8-T9 vertebra was made and a cord contusion of 100 kdynes was induced using an Infinite Horizon Impactor device (Precision System and Instrumentation; Lexington, KY, USA). This way, a transient, acute injury was inflicted to the spinal cord. The wound was sutured by planes and the animals allowed recovering in a warm environment. An intraperitoneal bolus of saline solution (B.Braun Vetcare, UK) was administered immediately after surgery. To prevent infection, amoxicillin (500 mg/L) (Normon, Spain) was given in the drinking water for one week. Postoperative analgesia was provided with buprenorphine (0.05 mg/kg) (B.Braun Vetcare, UK) for 48 h. Bladders were expressed twice a day until reflex voiding was re-established.

#### 2.7.1. Cell Administration

To perform the intrathecal administration, under ketamine/xylazine-anaesthesia, the vertebral column was exposed after muscle dissection at L3–L4 vertebrae, and two injections of 7.5 µL each of a suspension of 1 × 10^6^ MSC,WJ were slowly administrated using a Hamilton syringe with a 33-gauge needle into the cerebrospinal fluid between vertebras. Appropriate access to the intrathecal space was confirmed by animal tail flick. In each injection, the needle was held in place at the injection site for one additional minute to avoid reflux. After the injections, muscle and skin were sutured [21]. In total, four experimental groups of rats were studied: (i) one group (n = 5) received intrathecally vehicle (vehicle control, VHC) at 7 days post-lesion (dpl), (ii) a second group (n = 5) was injected intrathecally with a suspension of MSC,WJ at 7 dpl, (iii) a third group (n = 5) received two cell injections at 7 and 14 dpl, and (iv) a group of rats (n = 5) was injected with a suspension of MSC,WJ at 7 dpl and were culled at 9 dpl or 2 days post-injection (dpi), in order to assess the persistence of cells at the administration site, whereas the first three groups were followed up to 70 dpl. In order to prevent xenoreaction to human cells, a subcutaneous injection of FK506 was administered immediately after the transplantation (2 mg/kg), and additional injections of FK506 (1 mg/kg) were given to all animals in each experimental group once a day until the end of the follow-up period.

#### 2.7.2. Functional Assessment

Motor behaviour was tested before surgery and at 3, 7, 14, 21, 28, 35, 42, 49, 56, 63 and 70 dpl by open-field locomotion. Animals were placed individually in a circular enclosure and allowed to move freely for 5 min. Two observers evaluated locomotion during open-field walking and scored the hind limb performance according to the Basso, Beattie, Bresnahan (BBB) scale [22]. Briefly, the BBB scale consists of an ordinal scale from 0 points (no discernible hind limb movement) to 21 points (consistent, coordinated gait with parallel paw placement of the hind limb and consistent trunk stability), and to the BBB-subscale [23]. The scale (0–21) represents sequential recovery stages and categorizes combinations of rat joint movement, hind limb movement, step, forelimb and hind limb coordination, trunk position and stability, paw placement, and tail position. To calculate BBB subscores, individual categories of BBB outcomes were quantified as previously indicated [23]. Each category within the subscores indicates whether independently specific aspects of locomotion are altered by the treatment (maximum score = 13).

#### 2.7.3. Electrophysiological Tests

For the electrophysiological tests, the animals were anesthetized at 70 dpl with ketamine (90 mg/kg) and xylacine (10 mg/kg), placed prone onto a metal plate and skin temperature maintained above 32°C. An electromyography apparatus (Sapphyre 4ME, Vickers Healthcare Co., Woking, UK) was used. Motor evoked potentials (MEPs) were elicited by transcranial electrical stimulation delivered by two needle electrodes placed subcutaneously over the skull, with the anode over the sensorimotor cortex and the cathode at the nose. Single electrical pulses of supramaximal intensity (25 mA, 100 μs) were applied, and the MEPs were recorded with needle electrodes from *tibialis anterior* (TA) and *gastrocnemius medialis* (GM) muscles. Peripheral motor nerve conduction (MNC) tests were performed by stimulating the sciatic nerve with single electrical pulses (100 μs at supramaximal intensity). The compound muscle action potential (CMAP, M wave) and the H-reflex wave were recorded from TA and *plantar interossei* (PL) muscles by means of needle electrodes. The recording active electrode was inserted on the belly of the corresponding muscle and the reference at the fourth toe. Motor evoked potentials (MEPs) were elicited by transcranial electrical stimulation delivered by two needle electrodes placed subcutaneously over the skull, with the anode over the sensorimotor cortex and the cathode at the nose. Single electrical pulses of supramaximal intensity (25 mA, 100 μs) were applied, and the MEPs were recorded with needle electrodes from the TA muscle. The latency and the onset-to-peak amplitude of the maximal M and H waves and MEP were measured. The H/M ratio was calculated to estimate the degree of hyperreflexia.

#### 2.7.4. Histology

At the end of the follow up period, at 70 dpl, rats were deeply anesthetized (pentobarbital, 100 mg/kg i.p.) and intracardially perfused with 4% paraformaldehyde (PFA; Sigma-Aldrich) in phosphate buffered saline (PBS). The spinal cord segment from 1 cm rostral to 1 cm caudal of the injury epicentre (2 cm total length) was harvested and postfixed in the same fixative solution for 2 h and cryopreserved in PBS supplemented with 30% (*w*/*v*) sucrose. For evaluation of the spared tissue, grafted area, and cell localization, longitudinal spinal cord sections (20 μm thick) were cut with a cryotome (Leica CM190; Leica Microsystems, Wetzlar, Germany) and distributed in 10 series of 8–9 sections (separated by 200 μm) each. Sections were collected onto gelatin-coated glass slides. Spinal cord sections were processed for immunohistochemistry with a specific antibody for human cells, Stem 101 against human nuclear protein (1:150; Clontech Takara Bio Inc., Mountain View, CA) to localize the engrafted cells. Other longitudinal sections were immunostained with a primary antibody against glial fibrillary acidic protein (GFAP; 1:500; Dako, Glostrup, Denmark) to visualize astroglial reactivity and the glial scar around the lesion. To label the immune cells recruited around the injury area, we used anti-Iba1 (1:500; Wako, Richmond, VA, USA), and neurofilament to label neurons and processes (RT-97, 1:200, Hybridoma Bank, Iowa City, IA). Spinal cord samples from the group of animals to follow-up the cells for 9 pdl were cut transversally (20 µm thick) and processed with a primary antibody against GFAP and a specific antibody for human cells, anti-mitochondria (Mito, 1:100; Abcam, Cambridge, UK) to localize the engrafted cells. Primary antibodies used are indicated in Supplementary Table S1.

Tissue sections were blocked with PBS supplemented with 0.3% Triton (Sigma-Aldrich) and 10% species-specific serum and incubated for 24 h at 4°C with the corresponding primary antibody diluted in PBS plus 0.3% Triton and 10% normal donkey serum (NDS; Sigma-Aldrich). After washes, sections were incubated for 2 h at room temperature with 1:200 dilution of secondary antibody (donkey anti-mouse Alexa Fluor 488 for Stem101, RT-97 and GFAP; donkey anti-rabbit Alexa Fluor 594 for GFAP; Thermo Fisher Scientific, Waltham, MA, USA) in conjunction with 4′,6-diamidino-2-phenylindole (DAPI; 1:1000; Invitrogen, Carlsbad, CA, USA). Slides were dehydrated and mounted with Fluoromount (Thermo Fisher Scientific). In all immunohistochemical procedures, we included internal controls (for primary and secondary antibodies) to detect nonspecific staining. Images were acquired under the same exposure time, sensibility, and resolution for each marker analysed with a digital camera (Nikon D5-Ri2) attached to the microscope (Nikon ECLIPSE Ni) and NIS-Elements BR software (version 5.11.03). Transversal section with engrafted cells were visualized and captured under scanning confocal microscopy (LSM 700 Axio Observer, Carl Zeiss, Germany). The areas of spared tissue, the cavity, and the total spinal cord section were delineated and measured using ImageJ software v1.72 (National Institutes of Health, NIH; Bethesda, MA, USA) on 16 longitudinal cord sections (separated by 200 μm) of each animal. Volume of spared tissue was calculated using the Cavalieri’s correction of morphometric volume [24].

### 2.8. Statistical Analysis

Data are shown as the mean ± standard error of the mean (SEM). Statistical comparisons between groups were made using two-way ANOVA with Tukey *post hoc* test in functional and histological studies. One-way ANOVA with Tukey *post hoc* test was used for the assessment of electrophysiology and spared tissue analysis. Finally, *t*-test analyses were used in cell proliferation and lymphocyte proliferation assay. Differences between groups were considered statistically significant at *p* < 0.05. Statistical analyses were performed with Prism 8 software (GraphPad, La Jolla, CA, USA).

## 3. Results

### 3.1. Characteristics of MSC,WJ

Identity of cells used in the present study was compliant with established product specifications, being negative for the expression of CD45, CD31, and HLA-DR; and positive for CD73, CD90, and CD105 surface markers (Table 2). Given that MSC,WJ were going to be used in an SCI model of immunocompetent animals treated with FK506 to avoid xenorejection of human cells, we first assessed potential interaction of this immunosuppressant with the identity and growth kinetics of MSC in culture.

**Figure 1:**
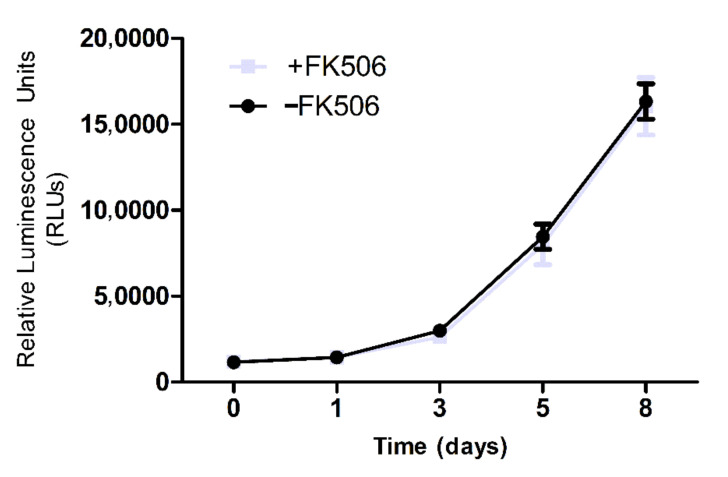
Growth profiles of MSC cultures in presence or absence of FK506. The growth of MSC,WJ in the two experimental settings is presented as the increase of ATP content, measured by luminescence.

Remarkably, no differences were observed in the growth curves studied in presence or absence of FK506 (Figure 1). Moreover, expression of surface markers of cultured cells was in accordance with established specifications (Table 3).

**Figure 2:**
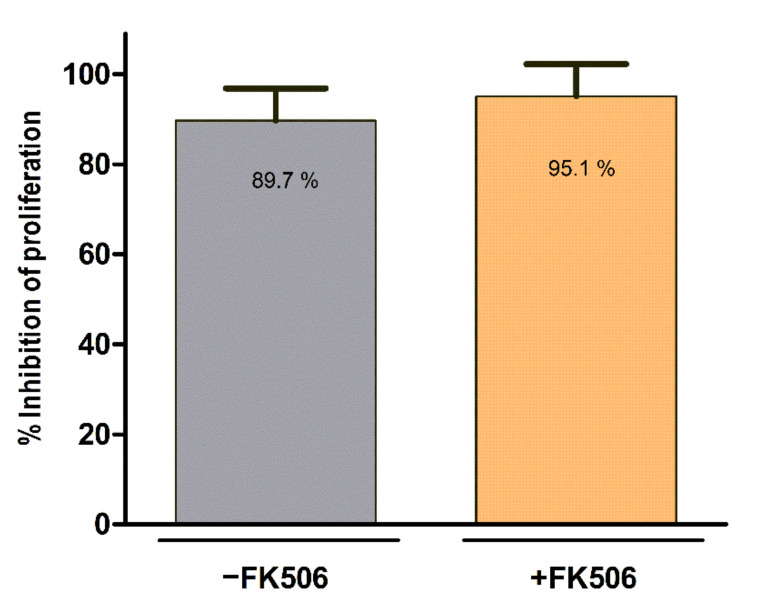
**Immunopotency assay for MSC in presence or absence of FK506.** Both conditions tested resulted in pronounced inhibition of the proliferation of stimulated NC in culture. Values of inhibition of proliferation in co-cultures of MSC,WJ:NC were normalized to proliferating stimulated NC in absence of MSC,WJ.

Immunomodulation potency of MSC cultured in presence or absence of FK506 was measured by their ability to inhibit the proliferation of polyclonally stimulated lymphocytes in vitro. The percentage of proliferating stimulated NC isolated from peripheral blood decreased drastically in co-cultures with MSC,WJ independently of FK506 treatment (Figure 2). Only a slightly higher percentage of inhibition of proliferation was observed when using FK506 in co-cultures (89.7 ± 7.1% vs. 95.1 ± 7.1%, in absence and presence of FK506, respectively). Once confirmed that identity and potency of MSC,WJ remained unaltered in the presence of FK506, we proceeded to conduct the in vivo studies in rat.

### 3.2. In Vivo Studies

#### 3.2.1. Toxicity of MSC,WJ Intrathecal Administration

To ensure the safety of the intrathecal administration of MSC,WJ, subchronic toxicity and biodistribution of the human cells were evaluated in athymic immunodeficient rats. To this end, animals receiving either an injection of a saline solution (control group, n = 3) or 10^6^ MSC,WJ (treated group, n = 9) were clinically evaluated for 3 months. Along this period, neither spontaneous mortality nor differences in food and water intake or in body weight were observed among control and treated animals. Despite some unspecific and non-clinically relevant ocular (chromodacryorrhea and/or ocular discharge) and respiratory symptoms (scratching and sneezing) were detected in both experimental groups with independence of the treatment received, all animals presented a normal and healthy general condition throughout the course of the study. During the performance of the macroscopic necropsy, unspecific alterations such as the presence of a hard white structure inside the urinary bladder or a local whitish area in the surface of the spleen were found in some control and treated rats (Supplementary Figure S1). Histologically, some clinically irrelevant lesions were described in both control and experimental groups. These observations included: (i) a minor accumulation of inflammatory cells in gastric mucosa, liver, pancreas, kidneys, duodenum, salivary glands and heart; (ii) a multifocal hyperplasia of Bronchus-Associated Lymphoid Tissue (BALT); and (iii) a mild fibrosis in the capsule of the spleen (Supplementary Figure S1). As intrinsic characteristic of the athymic strain, a lymphoid depletion of the T-dependent zones in secondary lymphoid organs was also seen in all animals. However, as mentioned above, none of these findings was associated to a pathology and could not be attributed to the administration of MSC,WJ. Regarding biodistribution, persistence of human cells was not detected in any of the analysed tissues at 3 months post-administration.

#### 3.2.2. Effects of MSC,WJ on Functional Recovery after SCI

**Figure 3:**
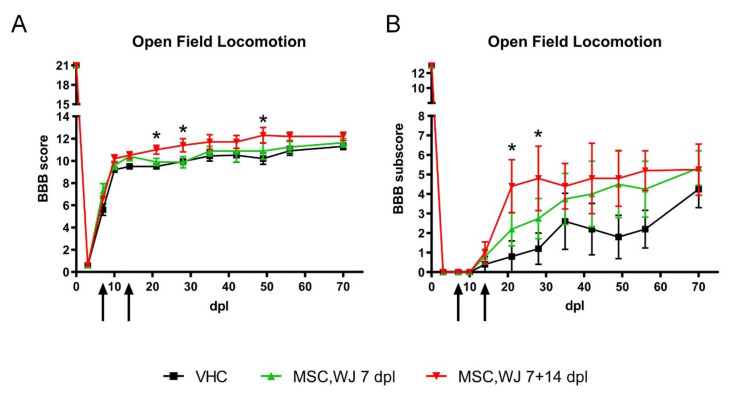
Effects of MSC,WJ administration on functional recovery. Open-field locomotion was evaluated weekly after spinal cord injury (SCI) using the score (**A**) and subscore (**B**) of the Basso, Beattie, Bresnahan (BBB) scale in the three studied groups (each group n = 5): vehicle or VHC (group of animals with SCI and vehicle administered intrathecally at 7 days post-lesion, or dpl), MSC,WJ 7 dpl (group of animals with SCI and 1 × 10^6^ MSC,WJ cells intrathecally at 7 dpl, arrows) and MSC,WJ 7 + 14 dpl (group of animals with SCI and 1 × 10^6^ MSC,WJ cells intrathecally at 7 and 14 dpl, arrows). All animals showed a temporal paralysis (BBB score of 0) just after the injury with partial recovery until day 14 and a plateau phase until the end of follow-up. * *p* < 0.05 group MSC,WJ 7 + 14 vs. VHC.

All animals were functionally tested at 1 and at 3 dpl to ensure the SCI had been correctly performed and were randomly distributed between the experimental groups. The BBB open-field locomotion score was used to test voluntary locomotion after SCI. Partial recovery was observed in all injured rats within the 2 weeks post lesion. From the second week, animals achieved a *plateau* of BBB scores of 10–12 points, indicating weight support and occasional/frequent plantar stepping performance. During the mid- and late-phases, a slightly higher BBB score was observed for MSC,WJ 7 + 14 group, showing significant differences (*p* < 0.05) at 21, 28 and 49 dpl compared to the vehicle and MSC,WJ 7 groups (Figure 3A). In the BBB subscale, that measures fine walking ability (from 0 to 13), both cell-administered groups reached higher scores than the vehicle group from 21 dpl, with significant differences from MSC,WJ 7 + 14 group at 21 and 28 dpl compared to the other two groups (Figure 3B).

**Figure 4:**
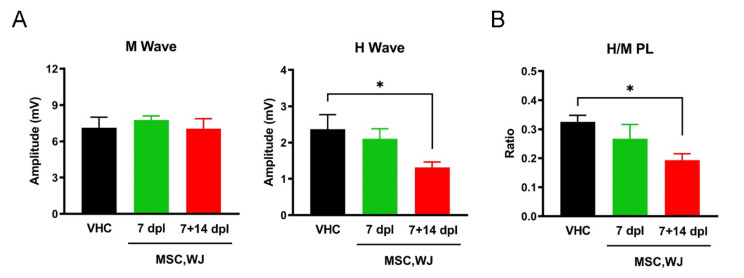
Motor nerve conduction studies at 70 dpi. (**A**) Amplitude of M and H waves recorded in the plantar muscles. The H wave was significantly reduced in MSC,WJ 7 + 14 days post-lesion (dpl) vs. vehicle (VHC). (**B**) Spinal reflex H/M ratio of plantar muscle showed a significant decrease in MSC,WJ 7 + 14 dpl group vs. VHC. (* *p* < 0.05). n = 5 per group.

Electrophysiological tests were performed at the end of the experiment (70 dpi). MNC tests showed normal values of the peripheral nerve conduction, without differences in latency and amplitude of the CMAPs between groups. However, injured animals transplanted with MSC,WJ at 7 + 14 dpl showed a significant decrease (*p* < 0.05) in the H/M ratio of the PL muscle, indicative of reduced spinal cord excitability and hyperreflexia, compared to control vehicle animals (*p* < 0.05) (Figure 4). MEPs were recorded as small polyphasic potentials of delayed latency, as expected after a SCI [25], indicating preservation of a limited amount of descending motor pathways. MEPS were recorded in the TA muscle of 3 of 5 rats of group vehicle (mean amplitude 83 ± 49 µV), and in 4 of 4 rats tested of groups MSC,WJ 7 and MSC,WJ 7 + 14 (718 ± 384 µV and 622 ± 220 µV, respectively).

### 3.3. Histological Results

#### 3.3.1. MSC,WJ Localization after Intrathecal Administration

**Figure 5:**
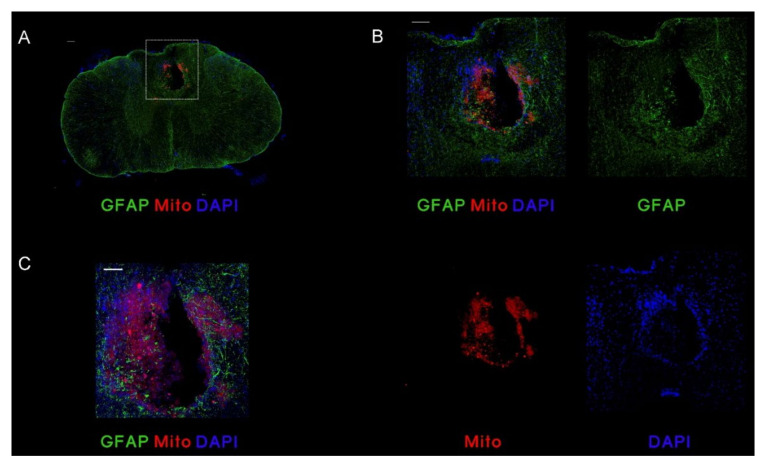
MSC,WJ localization after intrathecal administration at 9 days post-lesion (dpl) and 2 days post-injection (dpi). Representative images of rats with transplanted MSC,WJ. Cells were injected intrathecally (L3–L4). After 2 days, staining for Mito was observed inside the lesion site. (**A**) Representative microphotography of transversal section showing injured spinal cord at 9 dpl after 2 dpi intrathecal injection of MSC,WJ. (**B**) Magnification of injured spinal cord (from white box on A) showing GFAP staining in green, Mito staining in red and 4′,6-diamidino-2-phenylindole (DAPI) staining in blue. MSC,WJ can be identified into the lesion site. (**C**) Confocal microphotography shows localization of engrafted cells at lesion site. Bar scale: 100 µm. n = 3.

**Figure 6:**
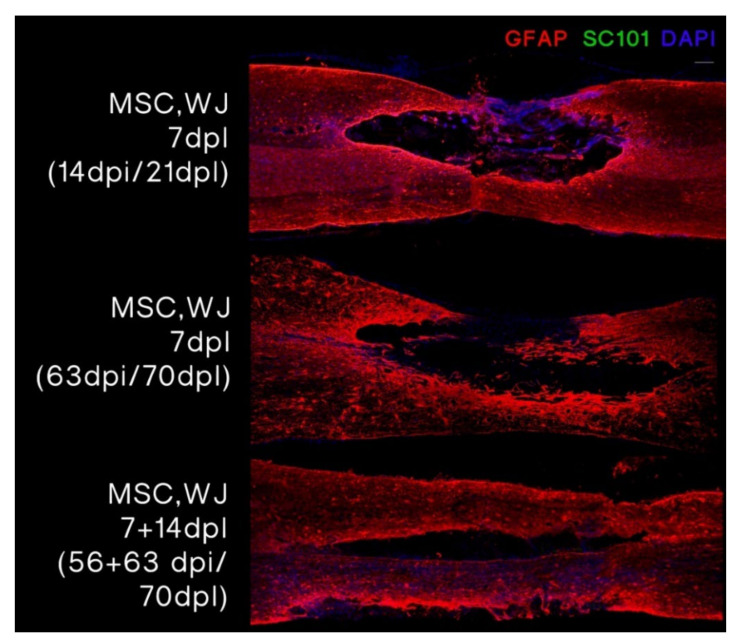
MSC,WJ localization after intrathecal administration at 21 or 70 days post-lesion (dpl), 14 days post-injection (dpi) or 63/56 + 63 dpi). Representative images of each group with transplanted MSC,WJ. No staining for SC101 was observed around or inside the lesion. GFAP staining in red, SC101 in green and DAPI in blue. Bar scale: 100 µm. n = 3.

We evaluated the area surrounding the lesion in all animals receiving cell treatment. MSC,WJ were labelled with human anti-mitochondria (Mito) in samples taken at day 2 post administration (9 dpl) and with human nuclear marker SC101 in samples taken at day 14 post administration (21 dpl) and at 56 and 63 days post administration (70 dpl). Remarkably, human cells were found at the lesion site at 2 dpi (9 dpl) (Figure 5) but were not detected at later time points (21 dpl and 70 dpl) (Figure 6).

#### 3.3.2. Spared Cord Tissue after SCI and Inflammatory Response

**Figure 7:**
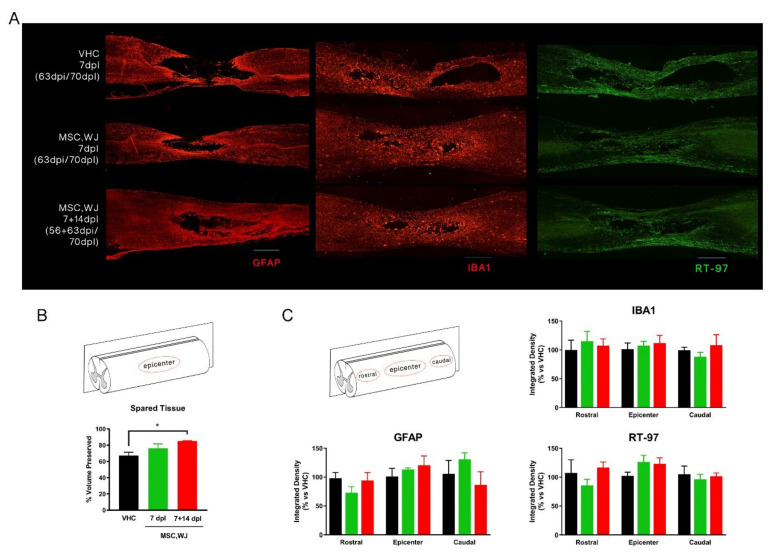
Histological studies. (**A**) Representative longitudinal images from each experimental condition. From left to right: Glial fibrillary acidic protein (GFAP) staining in red (marked astrocytic reactivity around the lesion), Iba1 staining in red (immune cell recruited around the injury), and RT-97 staining in green (axonal pathways within the injured spinal cord). Bar scale: 100 µm. (**B**) Scheme of measured areas in B. Quantification of estimated % volume of spared tissue. Significant increase of spared tissue was observed in MSC,WJ 7 + 14 dpl group vs. vehicle (VHC); * *p* < 0.05. (**C**) Scheme of measured areas in C. Measurements of immunolabeled density of microglia (Iba1), astroglial reactivity (GFAP) and axonal reactivity rostral, caudal and lesion site (epicenter) at 70 days post-lesion (dpl) did not reveal changes induced by MSC,WJ administration cells. Scale bar: 100 μm. n = 5 in each condition.

We evaluated the amount of spared tissue at 70 dpl in longitudinal sections of the spinal cord by GFAP immunostaining. A glial scar was found surrounding the injured area and clearly delimiting spared tissue from the cystic cavity. The amount of preserved tissue was quantified by measuring the area of the cavity in each slide and estimating the total volume for the whole spinal cord. Interestingly, a larger volume of spared cord tissue was observed in animals treated with MSC,WJ and statistical significance was found when comparing the experimental group receiving dual dose versus the control group (Figure 7A,B). Immunohistochemical staining against neurofilaments showed RT-97 positive labelling along the spinal cord injured area forming an extensive network of axonal pathways, with slight though not significant higher levels at the epicentre in the groups injected with MSC,WJ (Figure 7A,C). Regarding astroglial reactivity, no differences were detected between groups (Figure 7C). Finally, there was intense recruitment of microglia and monocytes, labelled with anti-Iba1, at the injury site, but no differences were detected between groups (Figure 7A,C).

## 4. Discussion

MSC are self-renewing, ex vivo culture-expandable stem cell populations derived from the stroma of virtually all vascularized tissues of the body, because of their origin from perivascular multipotent progenitors [26]. The ease of their expansion up to clinically relevant numbers for therapeutic use makes them attractive for testing in a variety of conditions requiring modulation of inflammation or management of the dysregulated immune system through the secretion to the microenvironment of molecules with paracrine activity [27,28]. Their use in the allogeneic context benefits from these cells being immune privileged and it is associated to an excellent safety profile regardless of tissue source, dose, and route of administration [29,30]. Moreover, MSC play important roles in the homeostasis of tissues and response to injury. Despite of the variety of tissue sources, all MSC share similar properties in terms of regulation of immune responses and variability observed by different laboratories is most likely due to (i) the lack of standardization of MSC production protocols and (ii) diverse characterisation methods for defining identity, purity and potency. In this context, MSC from the umbilical cord is overtaking other traditional sources (i.e., bone marrow, lipoaspirates) mainly due to easy access and abundance of donations, straightforward derivation methods and the lack of ethical concerns [5,31]. Accordingly, we have successfully established a master bank of MSC,WJ and scaled up production for use in clinical trials [32]. To date, we have tested these cells in terms of safety and evaluated signs of efficacy in the context of inflammatory conditions, including chronic spinal cord injury (EudraCT No. 2015-005786-23; ClinicalTrials.gov Id. NCT03003364) and severe respiratory distress due to SARS-CoV-2 infection (EudraCT No. 2020-001505-22; ClinicalTrials.gov Id. NCT04390139) [33,34]. MSC,WJ used in the present study were in compliance with both the specifications established in the IMPD and the minimal criteria established by the International Society for Cell and Gene Therapy [35]. Provided that FK506 was used in the in vivo study, we considered important to evaluate the potential drug interaction with MSC,WJ in case the immunosuppressant had any effect on the qualities of the cell-based medicine under study. Importantly, critical quality attributes (identity and potency) of MSC,WJ were not affected by the presence of FK506.

The dose used in the present in vivo studies (1 × 10^6^ MSC) is in accordance to those reported in Oliveri’s meta-analyses (ranging from 10^3^ to 9 × 10^6^ MSC) [11], and to the dose proposed in the clinical study currently recruiting patients with chronic traumatic cervical incomplete SCI (1 × 10^6^ MSC,WJ/Kg; EudraCT No. 2021-000346-18; Clinicaltrials.gov Id. NCT05054803). With respect to biodistribution, our results are in line with studies performed by other authors showing a rapid clearance of cells after injection and no presence of cells (locally or systemically) at mid/long term after intrathecal administration [36,37].

Regarding the in vivo pilot study, we examined the effects of intrathecal administration of MSC, WJ in animals with SCI caused by controlled contusion at the thoracic level, the SCI model best mimicking most traumatic cases in humans [38]. Three groups of rats were studied, in which vehicle or a suspension of MSC,WJ was injected intrathecally at 7 dpl, and a third group received two cell injections at 7 and 14 dpl. We examined the effects of intrathecal MSC,WJ administration on functional recovery after SCI during 70 days. The animals were subjected to an anaesthetic procedure for the intrathecal administration, all of them at day 7 post-lesion and one group also at day 14 post-lesion, and they were subjected to subsequent surgical procedure and intrathecal injection. Despite these procedures, we did not observe any deterioration in the functional follow up.

All the rats achieved a score between 10 and 12 in the BBB scale, as expected, indicating weight support and occasional/frequent plantar stepping performance. The experimental group with two doses of MSC,WJ showed significant improvement in locomotion with respect to the other two groups, just one week after second administration (21 dpl), and this improvement was maintained at day 28 post lesion. Fine walking ability examined with the BBB subscale, also showed significant improvement in the experimental group receiving two doses of MSC,WJ at 21 and 28 dpl, as well as a trend to higher scores compared to the vehicle control group during the follow-up. Moreover, MSC,WJ at 7 + 14 dpl showed a significant decrease in the H/M ratio of the PL muscle, indicative of reduced hyperreflexia suggesting sparing of corticospinal and other descending pathways that influence the reflex arc. The histological results also indicated an increase in the volume of spared spinal cord tissue in the group of animals treated with two doses of MSC,WJ, as observed in GFAP staining and volume measurement of spared tissue. The higher effects of the two doses compared with a single dose of intrathecally injected MSC,WJ is in agreement with previous studies in which we found that doubling the cell dose of MSC injected within the injured spinal cord, significantly improved locomotion recovery and tissue protection in SCI rats [39]. In addition, administration of immunosuppressive FK506 improves the survival of grafted cells, enlarging the time window during which the grafted cells can act and release protective factors [39]. Thus, a higher amount of MSC transplanted plus temporary immunosuppressive treatment may be a feasible strategy to improve the outcomes in regenerative therapies for SCI.

We also examined the localization of MSC,WJ into the spinal cord. MSC,WJ migrated intrathecally to the lesion site (at T8-T9), at least 2 dpi, but we could not detect the presence of cells in any of the treated animals from 14 days after intrathecal injection. Other studies could not detect bone marrow-derived MSC in injury site after intrathecal administration either in contrast with other cell types as neural stem/progenitor cells (NSPCs) that migrate to the lesion site [39]. Other studies using in vivo luminescence imaging showed that a few of the transplanted luciferase-labelled human umbilical cord MSC tended to migrate towards the lesion epicentre [40]. Our findings strengthen a possible paracrine mechanism from MSCs. This may be due to the differential response of derived-MSC to chemokines that are released at the site of injury. Despite all studies that show their limited presence in cerebrospinal fluid, the beneficial effects of the repeated administration of MSCs suggest a paracrine effect mediated by the release of growth factors, anti-apoptotic molecules and anti-inflammatory cytokines, creating a favourable environment for neuroprotection. This possible paracrine mechanism could be responsible in the improvement of locomotion in the two-cell dose administration group, after the second injection. Furthermore, decreased hyperreflexia and increase of the volume of spared tissue can be directly related with the suggested paracrine effect of MSC.

In addition to the scientific significance of the work presented in this manuscript in terms of safety and efficacy of intrathecal administration of MSC,WJ in a contusion model of SCI in rats, we provided information on its relevance in the context of a broader development programme we are conducting in order to support future marketing authorisation application of an academic advanced therapy medicinal product in Europe. In order to reduce variability both in the preclinical studies and the clinical trials, we paid special attention to generate large master cell banks of MSC,WJ that would guarantee the sourcing of sufficient batches of cells at all stages of product development. In particular, we previously estimated that our bioprocess design holds the potential to yield 1920 total doses of 50 × 10^6^ cells from a single fragment of UC, after a maximum of 37.3 cumulative population doublings (CPD) [16]. Moreover, the robustness of this bioprocess design has been demonstrated in terms of batch-to-batch consistency of Critical Quality Attributes (CQA) including identity, purity, potency, product stability and target dose [9]. In this sense, we showed the scope and the degree of compliance with pharmaceutical standards, which is a type of information barely found in the scientific literature but otherwise highly relevant to other developers embarking on such an endeavour.

## 5. Conclusions

Altogether our preclinical data suggest that MSC,WJ-based treatment in our rat model of SCI is safe, without adverse reactions, and promoted slight spared tissue and functional improvement. Results of the study are encouraging to proceed with multiple dose clinical trials in our product development program in line with novel treatment strategies that combine neuroprotective and neuroregenerative activities.

## Figures and Tables

**Table 1:** List of studies conducted in the development of experimental MSC,WJ-based therapy for the treatment of traumatic spinal cord injury. Compliance with specific quality standards is described in the left column. GxP = compliance with Good Scientific Practices; GLP = Good Laboratory Practice; GMP = Good Manufacturing Practice; GCP = Good Clinical Practice; PD = Pharmacodynamics; PK = Pharmacokynetics; SCI = Spinal Cord Injury.

GxP	Type	Description	Ref.
GMP	Production process and Product characterisation	Validation of a bioprocess design for the derivation and scale up of human MSC,WJ for clinical use	[9,16]
GLP	Safety in vitro	Assessment of telomerase activity, proto-oncogene expression, G-banding karyotype and senescence of clinical grade human MSC,WJ	[16]
GLP	Safety in vivo	Biodistribution and toxicity assessment after administration of clinical grade human MSC,WJ in immunodeficient mice by intrathecal and tail vein injection	[16]
n/a	Product characterisation	Evaluation of potential interaction of FK506 and MSC,WJ in terms of viability, identity, proliferation and immunopotency	This work
GLP	PK/Tox in vivo	Subchronic toxicity assessment and biodistribution of human MSC,WJ after intrathecal administration in immunodeficient rats	This work
n/a	PD/PK in vivo	Evaluation of the effect and persistence of intrathecal administration of clinical grade human MSC,WJ in chronic traumatic model of SCI in rat	This work
GCP	Phase I/IIa	Randomized double-blind, crossover, placebo-controlled phase I/IIa clinical trial in 10 patients (7 males, 3 females, age range: 25–47 years) with chronic complete SCI (AIS-A) at dorsal level (T2-T11) (1 dose 1 × 10^7^ MSC,WJ).	[12] EudraCT No. 2015-005786-23; Clinicaltrials.gov Id. NCT03003364
GCP	Phase I/IIa	Randomized double-blind, placebo-controlled phase I/II clinical trial in patients with chronic (1 to 5 years) incomplete cervical lesion (2 doses 1 × 10^6^ MSC,WJ/Kg)	EudraCT No. 2021-000346-18; Clinicaltrials.gov Id. NCT05054803

**Table 2:** Immunophenotype of human MSC,WJ used in this study was in accordance with specifications established in the approved IMPD.

	Acceptance Criteria	Values (%)		
Viability	≥70%	97.2		
Identity	Positive (≥95%) for CD 105, CD 90, CD 73	CD105 99.8	CD90 99.9	CD73 99.8
	Negative (≤5 %) for CD 45, CD 31, HLA-DR	CD45 0.0	CD31 0.0	HLA-DR 0.0

**Table 3:** Viability and immunophenotype of cells after 8 days in culture in the presence or absence of FK506.

	Acceptance Criteria	Values (%) (−FK506/+FK506)		
Viability	≥70%	95.7/93.9		
Identity	Positive (≥95%) for CD 105, CD 90, CD 73	CD105 99.5/99.8	CD90 99.9/99.8	CD73 99.5/99.5
	Negative (≤5 %) for CD 45, CD 31, HLA-DR	CD45 0.0/0.0	CD31 0.0/0.0	HLA-DR 0.0/0.0

## Data Availability

All data are presented in the manuscript and the supplementary materials.

## Supplementary Materials

The following supporting information can be downloaded at: https://www.mdpi.com/article/10.3390/cells11142153/s1, Figure S1: Assessment of subchronic toxicity and biodistribution of MSC,WJ in immunodeficient rats.; Table S1: Antibodies used for immunolabeling of histological sections.

## Abbreviations

BALT	Bronchus-Associated Lymphoid Tissue
BBB	Basso, Beattie, Bresnahan
CFSE	carboxy–fluorescein diacetate succinimidyl ester
CMAP	compound muscle action potential
CPD	Cumulative Population Doublings
CQA	Critical Quality Attributes
DAPI	4′,6-diamidino-2-phenylindole
DMEM	Dulbecco’s Modified Eagle’s Medium
Dpi	days post-injection
dpl	days post-lesion
GM	gastrocnemius medialis
GMP	Good Manufacturing Practice
HBV	hepatitis B virus
HCV	hepatitis C virus
HIV	human immunodeficiency virus
hSer	human serum
IMPD	Investigational Medicinal Product Dossier
MEP	Motor evoked potential
MNC	motor nerve conduction
MSC	multipotent Mesenchymal Stromal cells
NAD	nucleic acid detection
NC	nucleated cells
NDS	normal donkey serum
NIH	National Institutes of Health
OECD	Organisation for Economic Co-operation and Development
PBS	phosphate buffered saline
PD	pharmacodynamics
PFA	paraformaldehyde
PK	pharmacokinetics
PL	plantar interossei
PMA	Phorbol 12-myristate 13-acetate
SCI	Spinal Cord Injury
SEM	standard error of the mean
TA	tibialis anterior
Tox	Toxicology
WJ	Wharton’s jelly
