# Supplementary material for: Preclinical Development of a Therapy for Chronic Traumatic Spinal Cord Injury in Rats Using Human Wharton’s Jelly Mesenchymal Stromal Cells: Proof of Concept and Regulatory Compliance
Source: Cells. 2022 Jul 8;11(14):2153. doi: 10.3390/cells11142153 (PMC9319990; doi:10.3390/cells11142153)
Supplement: Supplementary file 1 [file cells-11-02153-s001.zip › cells-1788604-supplementary.pdf]

cells MDPI

Supplementary Materials

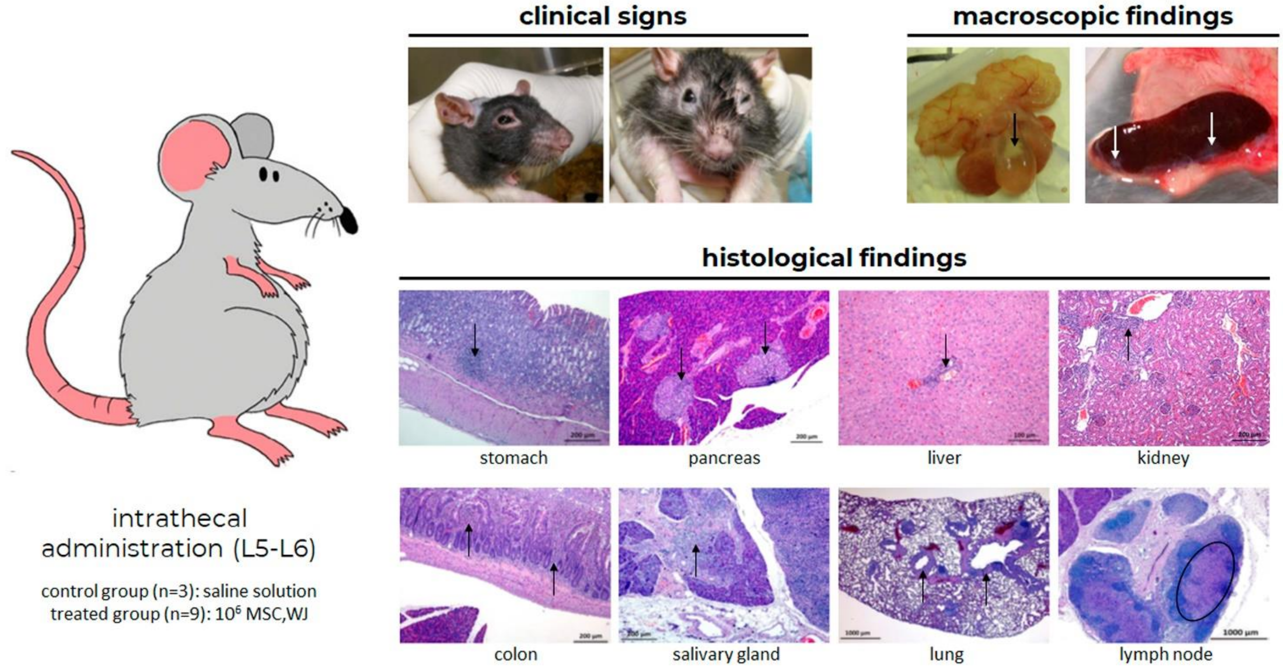

Supplementary Figure S1. Assessment of subchronic toxicity and biodistribution of MSC,WJ in immunodeficient rats. Athymic rats were administered either with saline solution (n=3, control) or 1 × 10^6 MSC (n=9, test group) intrathecally between vertebrae L5-L6 and culled at 3 months post-administration. No differences were observed between the test and control groups, and the only findings at clinical, organ macroscopic and histological levels are depicted in representative images showing: i) irrelevant and unspecific ocular symptoms observed during the clinical follow-up; ii) macroscopic alterations identified during the necropsy, such us the presence of a hard white structure inside the urinary bladder (black arrows) and a local whitish area in the surface of the spleen (white arrows); and iii) histological findings which include the minor accumulation of inflammatory cells in gastric mucosa, pancreas, liver, duodenum or salivary glands (black arrows), a multifocal hyperplasia of BALT in lungs (black arrows), and a lymphoid depletion of the T-dependent zones in lymphoid organs (black circle), characteristic of the athymic strain. All these alterations were observed in animals from both experimental groups and cannot be attributed to the administration of MSC, WJ.

Supplementary Table S1. Antibodies used for immunolabeling of histological sections.

Marker	Description	Target Cell	Host	Dilution	Company
RT-97	Neurofilament-H	Neuron cells	Mouse	1:200	Hybridoma Bank
GFAP	Glial fibrilary acidic protein	Glial cells	Rabbit	1:500	Dako
Iba1	Calcium binding protein	Microglia/macrophages	Rabbit	1:500	Wako
Stem101	Human nuclear protein	Human cells	Mouse	1:500	Clontech Takara
Mito	anti-mitochondria	Human cells	Mouse	1:100	Abcam
